# Complete Genome Sequence of an NADC30-Like Porcine Reproductive and Respiratory Syndrome Virus Characterized by Recombination with Other Strains

**DOI:** 10.1128/genomeA.00330-16

**Published:** 2016-05-05

**Authors:** Yingying Li, Guobiao Ji, Juan Wang, Feifei Tan, Jinshan Zhuang, Xiangdong Li, Kegong Tian

**Affiliations:** a College of Animal Science and Veterinary Medicine, Henan Agricultural University, Zhengzhou, People’s Republic of China; b National Research Center for Veterinary Medicine, High-Tech District, Luoyang, People’s Republic of China; c Veterinary Diagnosis Center and OIE Porcine Reproductive and Respiratory Syndrome Laboratory, China Animal Disease Control Center, Chaoyang District, Beijing, People’s Republic of China

## Abstract

We report here the complete genome sequence of an NADC30-like porcine reproductive and respiratory syndrome virus (PRRSV), HNyc15, which was characterized by recombination with VR-2332 and CH-1a PRRSV strains in open reading frames (ORFs) 2 to 4.

## GENOME ANNOUNCEMENT

Porcine reproductive and respiratory syndrome virus (PRRSV) is a single-stranded RNA virus belonging to the family *Arteriviridae* (1, 2). Since its emergence in the late 1980s, it has been recognized as an important threat that has caused great economic losses to the swine industry worldwide (3, 4). PRRSV can be divided into genotype 1 (European-like; prototype strain Lelystad) and genotype 2 (North American-like; prototype strain VR-2332), which share about 60% nucleotide identity at the genome level. Recently, several NADC30-like PRRSVs were isolated in China, which show the highest nucleotide similarity to a group of PRRSVs represented by NADC30, a type 2 PRRSV that was isolated in the United States in 2008. These NADC30-like PRRSV strains include Henan-XINX (GenBank accession no. KF611905), Henan-HEB (accession no. KJ143621), and JL580 (accession no. KR706343). Here, we report the complete genome sequence of another NADC30-like PRRSV, HNyc15, which was isolated from diseased pigs in Henan Province, China, in 2015.

The virus was isolated from a hilar lymph node sample from a piglet that showed clinical respiratory syndromes. To determine its complete genome sequence, 12 pairs of oligonucleotide primers were used to amplify different regions of the HNyc15 genome by reverse transcription-PCR (RT-PCR) (5). The PCR products were purified and cloned into pGEM-T Easy vector (Promega) and were sequenced with an automated sequencer (Genetic Analyzer 3730xl; Applied Biosystems). The complete genomic sequence of HNyc15 is 15,047 nucleotides (nt) long, with a 5′ untranslated region (UTR) of 190 nt, followed by a polyprotein precursor coding sequence and a 3′ UTR of 151 nt. Open reading frame 1a (ORF1a) and ORF1b of the virus are 7,116 and 4,374 nt in length, respectively, and ORFs 2 to 7 are 771, 765, 537, 603, 525, and 372 nt in length, respectively. Genetic analyses demonstrated that HNyc15 exhibits 61.2%, 85.2%, and 93.8% nucleotide identity with PRRSV strains Lelystad virus (LV), VR2332, and NADC30, respectively. Similar to other NADC30-like PRRSVs, HNjz15 exhibits a 1-amino acid (aa) deletion at position 303, a 111-aa deletion at positions 323 to 433, and a 19-aa deletion at positions 501 to 519, which can be used as molecular markers to distinguish NADC30-like PRRSVs from other type 2 PRRSVs.

The unusual genomic character of HNyc15 compared with that of other PRRSV variants is the unparalleled incidence of recombination with other strains of PRRSV. It demonstrated recombination with classical PRRSV strains VR-2332 (accession no. U87392) and CH-1a (accession no. AY032626) between ORF2 and ORF4, as analyzed by SimPlot version 3.5.1, with a 500-bp window sliding along the genome alignment (20-bp step size). The genome data of HNyc15 will be helpful for elucidating the molecular characteristics and evolutionary trends of PRRSV.

### Nucleotide sequence accession number.

The complete genome sequence of the HNyc15 strain is available in GenBank under the accession no. KT945018.
